# Health-related quality of life after vascular surgery and endovascular treatment in subjects with critical limb ischemia

**DOI:** 10.12669/pjms.36.5.2680

**Published:** 2020

**Authors:** Emced Khalil, Sedat Ozcan

**Affiliations:** 1 Dr. Emced Khalil Department of Cardiovascular Surgery, Ordu University Research and Education Hospital, Ordu, Turkey; 2 Dr. Sedat Ozcan Department of Cardiovascular Surgery. Canakkale 18 Mart University Faculty of Medicine, Canakkale, Turkey

**Keywords:** Peripheral arterial disease, Peripheral bypass surgery, Endovascular treatment, Quality of life

## Abstract

**Background & Objective:**

Revascularization of the target vessel and restoration of distal flow is critical not only to reduce mortality and morbidity but also improve health-related quality of life (HRQoL) in patients with critical limb ischemia. However, data concerning the impact of surgical bypass and percutaneous transluminal angioplasty (PTA) on HRQoL is limited. This study aimed to compare the impact of surgical bypass and PTA on HRQoL in subjects undergoing superficial femoral artery (SFA) or popliteal artery revascularization.

**Methods:**

Seventy-one subjects who underwent successful revascularization of the SFA or popliteal artery either with surgical revascularization or PTA were enrolled in this study. Three months after revascularization, all subjects underwent ankle-brachial index (ABI) measurement, 6-minute walking test and completed the Nottingham Health Profile (NHP) questionnaire. The NHP score differences (measured at the post-procedural 3^rd^ month) between subjects undergoing surgical or endovascular (PTA) revascularization subjects was the primary outcome measure of the study.

**Results:**

Both groups experienced significant improvements in ABI and 6-minute walking distance at post-procedure three months. NHP total scores of Part I and Part II at post-procedural six months were similar in the two groups. However, social isolation [77.98 (0 - 85) vs. 22.53 (0 - 100), p=0.002] and physical abilities [78.7 (30.31 - 87.7) vs. 54.47 (0 - 100), P=0.014] domain scores of the surgical revascularization group were significantly higher than that of the endovascular treatment group.

**Conclusion:**

This study shows that total scores obtained from the NHP questionnaire applied three months after revascularization of SFA stenosis are similar in subjects undergoing surgical revascularization or PTA. However, the social isolation and physical abilities domains of the NHP are significantly higher in subjects receiving surgical revascularization compared to those receiving PTA.

## INTRODUCTION

Peripheral arterial disease constitutes a major healthcare problem and is reported to be the 3^rd^ leading cause of atherosclerotic cardiovascular death –after coronary heart disease and stroke.^1^ Revascularization of the target vessel and restoration of distal flow is therefore critical in these patient, not only to reduce mortality and morbidity but also to improve health-related quality of life (HRQoL).^2^,^3^ Earlier studies have revealed that revascularization of the lower extremity leads to a significant reduction in bodily pain and improvement in physical functioning for patients with claudication and critical limb ischemia.^4^-^7^

Superficial femoral artery (SFA) is particularly prone to atherosclerotic vascular disease due to its localization, it passes through a fibromuscular canal which exposes the artery to significant forces of flexion, extension, shortening, and torsion(8).^8^ This unique location of the SFA and associated mechanical forces are responsible for the chronic vascular injury stemming from the cyclic deformation, strain, and cellular proliferation.^9^ Surgical bypass is the traditional first-line revascularization strategy in TASC II class D SFA occlusions, owing to the lower patency rates reported with percutaneous transluminal angioplasty (PTA).^10^ However, recent advances in endovascular technology enable high success rates, reaching and exceeding 80–90%, with PTA in TASC II C/D lesions.^11^ As such, some have reported comparable patency rates with surgical revascularization and PTA in SFA stenosis.^12^ However, data concerning the impact of these two revascularization strategies on HRQoL are limited.

This study aimed to compare the impact of surgical bypass and PTA on HRQoL measured by the Nottingham Health Profile (NHP) in subjects undergoing SFA revascularization (surgical vs PTA) for critical limb ischemia.

## METHODS

All consecutive patients with critical limb ischemia resulting from severe peripheral arterial disease of the SFA or popliteal artery who underwent successful revascularization, either with surgical revascularization or PTA, were enrolled in this single-center study. Successful revascularization was defined as ≥ 0.15 increase in postprocedural ABI compared to preprocedural ABI. Subjects with failed revascularization or those refusing to participate in the study were excluded from the study protocol. Written informed consent was obtained from all participants. The study was approved by the Institutional review board and was conducted in accordance with the Helsinki Declaration.

Demographic and clinical features of the participants were recorded before enrollment. PTA interventions were performed in a standard fashion by the same interventional team, either by the ante grade or retrograde approach according to the operator’s discretion. Accurate measurements of lesion length and vessel diameter were obtained by calibration techniques. Lesions were crossed with a hydrophilic guide wire and an angled, tapered catheter. Drug coated balloons competent with target vessel diameter were used for all PTA interventions. Bail-out stenting was reserved for lesions resulting in flow-limiting dissections after balloon angioplasty. All patients received a loading dose of 300 mg clopidogrel followed by 75 mg daily for four weeks, and were given aspirin and statin therapy on a long-term basis.

Polytetrafluoroethylene (PTFE) grafts or reversed auto genous saphenous vein grafts were utilized in in arterial by-pass surgeries. The majority of bypasses were between the common femoral artery and above-the-knee popliteal artery; whereas the tibial artery was the target for distal anastomosis in the rest of the cases. All patients undergoing surgical bypass received aspirin and statin therapy on a long-term basis.

All subjects underwent ABI measurement, 6-minute walking test, and NHP assessment three months after revascularization. The NHP is a patient-completed questionnaire used to determine and quantify perceived medical status.^13^ The NHP has been validated and demonstrated to be capable of discriminating the health-related differences between subjects in variety of medical conditions.^14^ The instrument yields an overall score, two domain scores, and 12 category scores. Part I consists of 38 yes/no items in six dimensions: pain, physical mobility, emotional reactions, energy, social isolation, and sleep. Part II of the profile consists of seven yes/no statements that determine the frequency of daily problems with paid employment, housework, family relationship, social life, sex life, hobbies, and holidays. The answers to the Turkish items are weighted, giving a range of possible scores from 0 to 100. Lower scores indicate the lack of problems within a domain, and higher scores are proportional to the severity of problems.^15^

The difference in NHP scores at the post-procedural 3rd month between subjects undergoing surgical or endovascular revascularization was the primary outcome measure of the study. The differences in ABI, and 6-minute walking distance between the groups were the secondary outcome measures.

### Statistical Analysis

All analyses were performed on SPSS v21 (SPSS Inc., Chicago, IL, USA). For the normality check, the Shapiro-Wilk test was used. Data are given as mean ± standard deviation or median (minimum - maximum) for continuous variables according to normality of distribution, and as frequency (percentage) for categorical variables. Normally distributed variables were analyzed with the independent samples t-test. Non-normally distributed variables were analyzed with the Mann Whitney U test. Repeated measurements were analyzed with the Wilcoxon Signed Ranks test or Friedman’s analysis of variance by ranks depending on the count of measurements. Pairwise comparisons were performed with the Bonferroni correction method. Inter-group comparisons of the amount of change in these variables were performed by comparing the differences with the Mann Whitney U test. Categorical variables were evaluated by using the Chi-square tests or Fisher’s exact tests. Two-tailed p-values of less than 0.05 were considered statistically significant.

### Ethical Approval

This study is conducted after the approval of ethical review board Ordu University, Turkey on January 30, 2020, Ref No. 34989324-772.02-E.248.

## RESULTS

A total of 71 subjects were enrolled in the study (mean age 65.02 ± 11.25 years, 86% male). Thirty patients underwent surgical revascularization through common surgical bypass (83.3% underwent femoro-popliteal bypass and 16.7% underwent femoro-tibial bypass). Forty-one subjects underwent PTA (88% with ante grade approach and 22% with retrograde approach). 14.65% of the subjects undergoing endovascular treatment received a stent. Demographic features and lesion characteristics of the study groups are presented in Table-I. The groups were similar with respect to the presence of the concomitant diabetes, hypertension, coronary artery disease, and smoking habit. The frequency of hyperlipidemia was higher in the endovascular treatment group compared to surgical revascularization group (41.46% vs. 16.68%, p=0.049). The distribution of TASC two and Rutherford classes of the lesions and their localizations were also similar in both groups.

**Table-I:** Summary of patients’ characteristics with regard to intervention type.

	Intervention		

	Surgery (n=30)	Endovascular (n=41)	p
Age (years)	67.87 ± 10.73	62.95 ± 11.29	0.069
Gender (Male)	26 (86.67%)	35 (85.37%)	1.000
Smokers	15 (50.00%)	17 (41.46%)	0.636
Diabetes Mellitus	17 (56.67%)	19 (46.34%)	0.536
Hyperlipidemia	5 (16.67%)	17 (41.46%)	0.049
Coronary Artery Disease	12 (40.00%)	20 (48.78%)	0.622
Chronic Renal Failure	1 (3.33%)	1 (2.44%)	1.000
Hypertension	24 (80.00%)	30 (73.17%)	0.701
***Rutherford Classification***			
2 & 3	16 (53.33%)	15 (36.59%)	0.213
4	11 (36.67%)	16 (39.02%)	
5	3 (10.00%)	10 (24.39%)	
***TASC II***			
B	7 (23.33%)	11 (26.83%)	0.902
C	14 (46.67%)	17 (41.46%)	
D	9 (30.00%)	13 (31.71%)	
***Localization***			
Only SFA	15 (50.00%)	20 (48.78%)	1.000
SFA + Popliteal	15 (50.00%)	21 (51.22%)	

Data are given as mean ± standard deviation for continuous variables and as frequency (percentage) for categorical variables.

**Fig.1:**
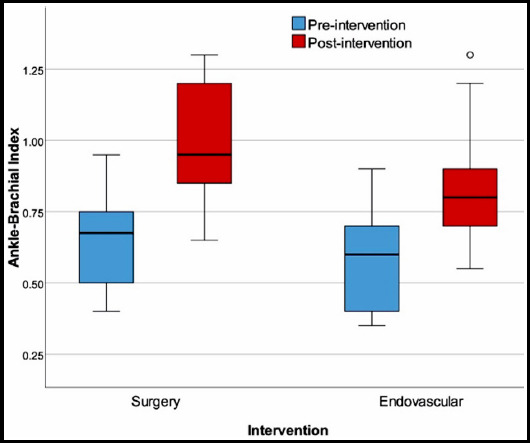
The change in ankle-brachial index from baseline to post-procedural 3 months with regard to intervention type.

**Fig.2:**
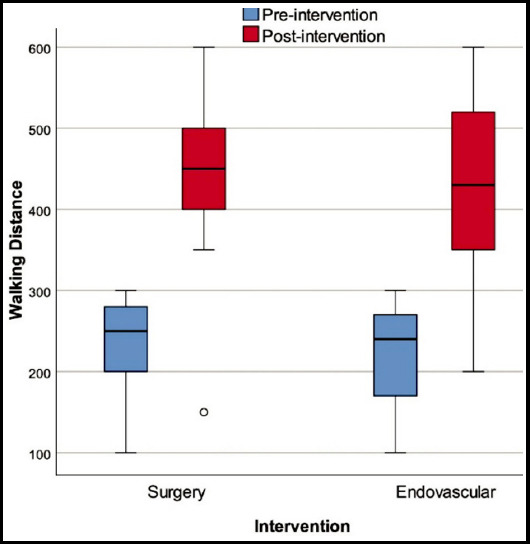
The change in 6-minute walking distance from baseline to the post-procedural 3 months with regard to intervention type.

Results of the three-month follow-up are presented in Table-II. Both groups experienced a significant improvement in ABI at post-procedural 3 months. The ABI values at the 3^rd^ month was significantly higher in subjects undergoing surgical revascularization compared to those undergoing endovascular treatment [0.95 (0.65 - 1.30) vs. 0.80 (0.55 - 1.30), p=0.033] (Fig.1). 6-minute walking distance significantly improved in both groups from baseline to the post-procedural measurement at six months. Walking distance at post-procedural six months was greater in the surgical revascularization group compared to the endovascular treatment group; however, this difference did not reach statistical significance [500m (150 - 600) vs. 420m (200 - 600), p=0.191] (Fig.2). NHP total scores from Part I and Part II at post-procedural 6 months were similar in the two groups. However, the social isolation [77.98 (0 - 85) vs. 22.53 (0 - 100), p=0.002] and physical abilities [78.7 (30.31 - 87.7) vs. 54.47 (0 - 100), P=0.014] domain scores of the surgical revascularization group were significantly higher than that of the endovascular treatment group.

**Table-II:** Summary of patients’ measurements and scale scores with regard to intervention type.

	Intervention		

	Surgery (n=30)	Endovascular (n=41)	p
***Ankle-Brachial Index***			
Pre-intervention	0.68 (0.40 - 0.95)	0.60 (0.35 - 0.90)	0.033
Post-intervention	0.95 (0.65 - 1.30)	0.80 (0.55 - 1.30)	
p (within groups)	<0.001	<0.001	
***Walking Distance***			
Pre-intervention	250 (100 - 300)	240 (100 - 300)	0.191
Post-intervention	500 (150 - 600)	420 (200 - 600)	
p (within groups)	<0.001	<0.001	
***Nottingham Health Profile (Part I)***			
Pain	76.6 (26.31 - 100)	69.77 (11.22 - 100)	0.777
Emotional reaction	62.75 (19.78 - 80.77)	64.56 (0 - 92.78)	0.177
Sleep	61.53 (21.7 - 87.27)	61.53 (0 - 100)	0.760
Social isolation	77.98 (0 - 85)	22.53 (0 - 100)	0.002
Physical abilities	78.7 (30.31 - 87.7)	54.47 (0 - 100)	0.014
Energy level	100 (0 - 100)	100 (0 - 100)	0.101
Total	460.12 (173.59 - 511.26)	367.01 (78.26 - 570.41)	0.057
***Nottingham Health Profile (Part II)***			
Work	11 (36.67%)	8 (19.51%)	0.180
Looking after the home	2 (6.67%)	5 (12.20%)	0.691
Social life	4 (13.33%)	12 (29.27%)	0.194
Home life	2 (6.67%)	4 (9.76%)	1.000
Sex life	6 (20.00%)	5 (12.20%)	0.509
Interests and hobbies	2 (6.67%)	7 (17.07%)	0.193
Vacations	5 (16.67%)	7 (17.07%)	1.000
Total	0 (0 - 6)	1 (0 - 6)	0.520

Data are given as median (minimum - maximum) for continuous variables and as frequency (percentage) for categorical variables.

## DISCUSSION

This study demonstrates that NHP total scores are similar in subjects undergoing surgical or PTA revascularization. However, the social isolation and physical abilities domains of the NHP showed significantly higher values in subjects receiving surgical revascularization. Both revascularization strategies led to significant improvements in ABI and 6-minute walking distance at post procedural measurement. These findings indicate that both revascularization strategies provide similar HRQoL in subjects undergoing revascularization of the SFA for critical limb ischemia.

A high proportion of subjects with peripheral artery disease experience leg pain during rest and exercise, which may, in turn, lead to ambulatory dysfunction and impaired physical functioning.^16^ Consequently, HRQoL is reported to be impaired in subjects with peripheral artery disease compared to healthy controls. Results from the population-based Edinburgh artery study indicated that people with intermittent claudication had impaired HRQoL as a consequence of reduced physical health; however, it was found that HRQoL was preserved in individuals with asymptomatic peripheral arterial disease.^17^ Although HRQoL is mainly a measure of self-perceived comfort, it may also be associated with poor survival. Moreover, some research has indicated that HRQoL scores of subjects with peripheral artery disease may be worse than individuals with coronary artery disease and congestive heart failure.^18^ For instance, Issa and colleagues have shown that HRQoL is an independent predictor of long-term survival in patients undergoing vascular surgery for peripheral artery disease, after adjusting for established prognostic factors.^19^

Critical limb ischemia is a debilitating disorder characterized by ischemic rest pain, non-healing ulcers and gangrene.^20^ In subjects with critical limb ischemia, impairment in HRQoL is more prominent compared to subjects with claudication.^21^ One of the earliest studies investigating HRQoL in subjects undergoing surgical revascularization, conservative treatment or major amputation for critical limb ischemia revealed that there were no significant differences between different treatment modalities in terms of HRQoL as measured by the SF-236 test.^22^ However, in that study, the authors did not analyze HRQoL score with respect to the type of the intervention. Similar to our results, Klevsgard and colleagues compared the improvement in NHP and SF-36 scores following successful revascularization of 80 subjects with claudication or critical limb ischemia and found that there were no significant differences in quality of life between patients who had undergone PTA and those who had undergone surgery.^4^ In a study that enrolled subjects with claudication or critical limb ischemia, Kalbaugh et al. reported that PTA was associated with excellent functional outcome despite the fact that PTA treatment demonstrated inferior patency rates compared to bypass.^23^

Although data concerning the post-revascularization improvements in HRQoL of subjects with claudication is extensive, evidence demonstrating the benefit of revascularization on HRQoL in subjects with critical limb ischemia is limited. One example to these studies is the research by Landry et al., who investigated functional status and quality of life in subjects undergoing revascularization for critical limb ischemia. They found that revascularization for critical limb ischemia caused improvements patient-perceived leg function. Similar to these findings, the Edifoligide for the Prevention of Infrainguinal Vein Graft Failure (PREVENT) III study reported improvement in HRQoL at 3 and 12 months after bypass was performed in the lower extremity for critical limb ischemia.^24^ Furthermore, the BASIL study (bypass versus angioplasty in severe ischemia of the leg) –one of the largest trials comparing surgical and endovascular treatment in terms of HRQoL in subjects with critical limb ischemia– reported that there was no difference in HRQoL between the surgical and endovascular treatment strategies.^25^

The present study shows that surgical bypass provides a better ABI at the 3^rd^ month of revascularization compared the PTA. However, both revascularization strategies provide similar improvements in 6-minute walking distance. Our results show that, except for the social isolation and physical abilities domains (which are more favorable in subjects undergoing PTA), subjects treated for critical limb ischemia with surgical bypass or PTA have similar HRQoL at3 months after revascularization. These findings are consistent with previous evidence and indicate that the improvements are similar in HRQoL with revascularization via surgical bypass or PTA for the treatment of critical limb ischemia.

### Limitations of the study

Firstly, and most importantly, NHP questionnaire results were not obtained before the revascularization procedure. Therefore, we cannot comment on whether the surgical revascularization and PTA provides significant improvement in HRQoL. Secondly, we provided single center data of a relatively small subject population. Further research with larger sample size is required to clearly address the role of surgical and endovascular revascularization strategies on HRQoL in subjects treated for critical limb ischemia.

## CONCLUSION

This study shows that total scores of the NHP questionnaire applied three months after revascularization of the SFA stenosis are similar in subjects undergoing surgical revascularization or PTA. Nevertheless, social isolation and physical abilities domains of the NHP are significantly higher in subjects receiving surgical revascularization than those receiving PTA. Both surgical and endovascular revascularization provides improvements in ABI and 6-minute walking distance at post procedural 3 months.

### Authors’ Contribution

**EK:** Conceived, designed and did statistical analysis & editing of manuscript. He also did review and final approval of manuscript and take responsibility of this study.

**EK & SO:** Did data collection and manuscript writing.

## References

[1] Murakami A (2018). Towards the Application of Endovascular Treatment for Superficial Femoral Artery. Cir J.

[2] Aber A, Lumley E, Phillips P, Woods HB, Jones G, Michaels J (2018). Themes that Determine Quality of Life in Patients with Peripheral Arterial Disease:A Systematic Review. Patient.

[3] Davie-Smith F, Coulter E, Kennon B, Wyke S, Paul L (2017). Factors influencing quality of life following lower limb amputation for peripheral arterial occlusive disease:A systematic review of the literature. Prosth Orthot Int.

[4] Klevsgard R, Froberg BL, Risberg B, Hallberg IR (2002). Nottingham Health Profile and Short-Form 36 Health Survey questionnaires in patients with chronic lower limb ischemia:before and after revascularization. J Vasc Surg.

[5] Bunte MC, Cohen DJ, Jaff MR, Gray WA, Magnuson EA, Li H (2018). Long-term clinical and quality of life outcomes after stenting of femoropopliteal artery stenosis:3-year results from the STROLL study. Catheter Cardiovasc Interv.

[6] Donker J, de Vries J, Ho GH, Goncalves FB, Hoeks SE, Verhagen HJ (2016). Review:Quality of life in lower limb peripheral vascular surgery. Vascular.

[7] Vlajinac H, Marinkovic J, Tanaskovic S, Kocev N, Radak D, Davidovic D (2015). Quality of life after peripheral bypass surgery:a 1-year follow-up. Wien Klin Wochenschr.

[8] Yahagi K, Otsuka F, Sakakura K, Sanchez OD, Kutys R, Ladich E (2014). Pathophysiology of superficial femoral artery in-stent restenosis. J Cardiovasc Surg (Torino).

[9] Banerjee S (2016). Superficial Femoral Artery Is Not Left Anterior Descending Artery. Circulation.

[10] Norgren L, Hiatt WR, Dormandy JA, Nehler MR, Harris KA, Fowkes FG (2007). Inter-Society Consensus for the Management of Peripheral Arterial Disease (TASC II). J Vasc Surg.

[11] Goltz JP, Kleemann M (2015). Complex recanalization techniques for complex femoro-popliteal lesions:how to optimize outcomes. J Cardiovasc Surg (Torino).

[12] Schmieder GC, Panneton JM (2008). Endovascular superficial femoral artery treatment:can it be as good as bypass?. Semin Vasc Sur.

[13] Hunt SM, McEwen J (1980). The development of a subjective health indicator. Sociol Health Illn.

[14] Hunt SM, McKenna SP, McEwen J, Backett EM, Williams J, Papp E (1980). A quantitative approach to perceived health status:a validation study. J Epidemiol Community Health.

[15] Kucukdeveci AA, McKenna SP, Kutlay S, Gursel Y, Whalley D, Arasil T (2000). The development and psychometric assessment of the Turkish version of the Nottingham Health Profile. Int J Rehabil Res.

[16] Andrade-Lima A, Cucato GG, Domingues WJR, Germano-Soares AH, Cavalcante BR, Correia MA (2018). Calf Muscle Oxygen Saturation during 6-Minute Walk Test and Its Relationship with Walking Impairment in Symptomatic Peripheral Artery Disease. Ann Vasc Surg.

[17] Dumville JC, Lee AJ, Smith FB, Fowkes FG (2004). The health-related quality of life of people with peripheral arterial disease in the community:The Edinburgh Artery Study. Br J Gen Pract.

[18] Regensteiner JG, Hiatt WR, Coll JR, Criqui MH, Treat-Jacobson D, McDermott MM (2008). The impact of peripheral arterial disease on health-related quality of life in the Peripheral Arterial Disease Awareness, Risk, and Treatment:New Resources for Survival (PARTNERS) Program. Vasc Med.

[19] Issa SM, Hoeks SE, Scholte op Reimer WJ, Van Gestel YR, Lenzen MJ, Verhagen HJ (2010). Health-related quality of life predicts long-term survival in patients with peripheral artery disease. Vasc Med.

[20] Brahmanandam SM, Messina LM, Belkin M, Conte MS, Nguyen LL (2009). PP44. Determinants of Hospital Disposition after Lower Extremity Bypass Surgery. J Vasc Sur.

[21] Treat-Jacobson D, Halverson SL, Ratchford A, Regensteiner JG, Lindquist R, Hirsch AT (2002). A patient-derived perspective of health-related quality of life with peripheral arterial disease. J Nurs Scholar.

[22] Hernandez-Osma E, Cairols MA, Marti X, Barjau E, Riera S (2002). Impact of treatment on the quality of life in patients with critical limb ischaemia. Eur J Vasc Endovasc Surg.

[23] Kalbaugh CA, Taylor SM, Blackhurst DW, Dellinger MB, Trent EA, Youkey JR (2006). One-year prospective quality-of-life outcomes in patients treated with angioplasty for symptomatic peripheral arterial disease. J Vasc Surg.

[24] Nguyen LL, Moneta GL, Conte MS, Bandyk DF, Clowes AW, Seely BL (2006). Prospective multicenter study of quality of life before and after lower extremity vein bypass in 1404 patients with critical limb ischemia. J Vasc Surg.

[25] Adam DJ, Beard JD, Cleveland T, Bell J, Bradbury AW, Forbes JF (2005). Bypass versus angioplasty in severe ischaemia of the leg (BASIL):multicentre, randomised controlled trial. Lancet.

